# Telemedicine-Based Digital Cognitive Behavioral Intervention for Perioperative Anxiety and Depression for Total Knee Arthroplasty

**Published:** 2023-09-27

**Authors:** Ata Murat Kaynar, Nicole Zharichenko, Ajay D Wasan, Jacques E Chelly

**Affiliations:** 1 Department of Anesthesiology and Perioperative Medicine, University of Pittsburgh Medical Center, Pennsylvania, USA; 2 Department of Anesthesiology and Perioperative Medicine, Center for Innovation in Pain Care (CIPC), Pennsylvania, USA; 3 Department of Critical Care Medicine, University of Pittsburgh, Pennsylvania, USA; 4 Department of Critical Care Medicine, Clinical Research Investigation and Systems Modeling of Acute Illness (CRISMA) Center, Pennsylvania, USA; 5 Department of Orthopedic Surgery, University of Pittsburgh, Pennsylvania, USA

**Keywords:** Anxiety, Depression, Telemedicine, Total Knee Arthroplasty (TKA), Patient-Reported Outcomes Measurement Information System (PROMIS)

## Abstract

**Introduction:**

Preoperative anxiety and depression have been shown to increase postoperative pain and opioid consumption by up to 50% in patients undergoing primary unilateral Total Knee Arthroplasty (TKA). We hypothesized that the use of a telemedicine-based digital Cognitive Behavioral Intervention program (RxWell^®^) started one month prior to surgery would control anxiety and depression prior to surgery.

**Materials and methods:**

This was a randomized, controlled trial that enrolled patients undergoing primary unilateral TKA. At least a month prior to surgery, patients who gave consent to participate were asked to complete PROMIS^®^ (Patient-Reported Outcomes Measurement Information System) emotional anxiety short form 8a and PROMIS^®^ emotional depression short form-8a questionnaires. Patients with T-scores of ≥ 57 were randomized to either a no intervention (control group) or a RxWell^®^ program (treatment group) for a month prior to surgery. The primary outcome of this proof-of-concept study was the ability of the RxWell^®^ to normalize patients’ PROMIS anxiety T scores.

**Results:**

T scores for anxiety and depression among patients randomized to the RxWell^®^ group significantly decreased from 64.3 ± 3.0 at the time of randomization to 58.5 ± 2.6 prior to surgery (n=5, p=0.006), whereas no changes in T scores were recorded in the control group (59.4 ± 4.2 at the time of randomization *vs*. 57.7 ± 6.2; n=6, p=0.559).

**Conclusion:**

These preliminary data suggest that the use of a RxWell^®^ program represents an effective approach to control anxiety and depression prior to surgery. In contrast, it seems that in the absence of treatment, anxiety level remains similar over a month prior to surgery.

## Introduction

Before surgery, approximately 15%−30% of patients undergoing Total Knee Arthroplasty (TKA) experience mood disorders, such as anxiety or depression [1]. While the surgery itself would decrease the postoperative frequency of anxiety and depression, these mood disorders negatively impact postoperative pain and opioid consumption, complications, recovery rate, and hospital re-admission [2–13]. Preoperative anxiety has been shown to be associated with an increase in postoperative pain and opioid consumption [14,15].

PROMIS^®^ (Patient-Reported Outcomes Measurement Information System) scores are standardized tools that allow researchers to explore mood disorders in the US population. These tests are a series of questionnaires that evaluate physical function, anxiety, and depression and have been validated across the US population. A T-score of 50 ± 10 is considered normal.

Although evidence suggests that Cognitive-Behavioral Intervention may be of value for preoperative control of catastrophizing, effective programs to control anxiety and depression remain to be established [16–22]. In this regard, telemedicine represents an increasingly common strategy for patient care. Lately, telemedicine has been utilized in more treatment scenarios such as remote psychotherapy. Similarly, we believe that digital Cognitive Behavioral Intervention (RxWell^®^) could help deliver Cognitive Behavioral Intervention (CBI) to a wider patient population. Preliminary data suggests that RxWell^®^ could be an effective treatment for anxiety and depression in primary care [23]. However, its value in surgical patients remains to be established. We hypothesized that the use of an RxWell^®^ program may be an effective tool to reduce anxiety and depression in patients undergoing primary and unilateral TKA [24].

## Materials and Methods

Our study was approved by the University of Pittsburgh Institutional Review Board and registered to the clinicaltrials.gov database (NCT05658796).

### Study design

This was a prospective, randomized, controlled clinical trial conducted in patients undergoing TKA at two UPMC (University of Pittsburgh Medical Center) hospitals (UPMC Shadyside and UPMC Passavant, Pittsburgh, PA). Patients were approached after completing a TKA clinical education program. After signing an informed consent form, each patient was asked to complete a PROMIS^®^ emotional anxiety short form 8a questionnaire and a PROMIS^®^ emotional depression short form-8a questionnaire. Patients with T scores of ≥ 57 were randomized to either a control group (no intervention) or treatment group involving being included in a mobile application (RxWell^®^) guiding patients with anxiety or depression through CBI learnings and techniques such as, relaxation, cognitive reframing, exposure, and mindfulness. It offers patients tele-access to a live coach *via* a text messaging component within the application. This remote experience helps guide and motivate the patient through the program and to apply the techniques in everyday life situations. The program required the patient of involvement for a minimum of 4 weeks prior to surgery.

### Statistical analysis

PROMIS^®^ emotional anxiety short form 8a and PROMIS^®^ emotional depression short form-8a scores at the time of enrollment and prior to surgery were compared using a two-tailed paired t-test in the control and treatment group. Alpha was set at 0.05 and data are presented as mean ± Standard Deviation (SD).

## Results

The study began in February 2023 and the present work is referring to a part of the approved protocol. 349 patients undergoing unilateral primary TKA were screened, of which 68 gave consent to participate. Fifteen patients were randomized (T score ≥ 57). Surgery was cancelled for two patients. One patient in the treatment group did not follow the RxWell^®^ program. Among the remaining 12 patients, three patients in each group completed the PROMIS^®^ emotional anxiety short form 8a questionnaire and two of the same patients in the RxWell^®^ group and three patients in the control group completed a PROMIS^®^ emotional depression short form-8a prior to surgery. The compliance was only 50% (6/12 completed both PROMIS scales at the time of enrollment and prior to surgery. The contributing factors for low compliance could be due to long time interval (one month) between enrollment and surgery in the intervention arm and lack of engagement in the control arm. Increased coaching and frequent reminders over telemedicine are being considered to increase compliance.

Overall anxiety and depression T-score in the patients who completed PROMIS^®^ emotional anxiety short form 8a and PROMIS^®^ emotional depression short form-8a questionnaires and whose score was >57 at the time of randomization and who completed the same questionnaires at prior to surgery was 61.5 ± 4.2.

**Figure 1:**
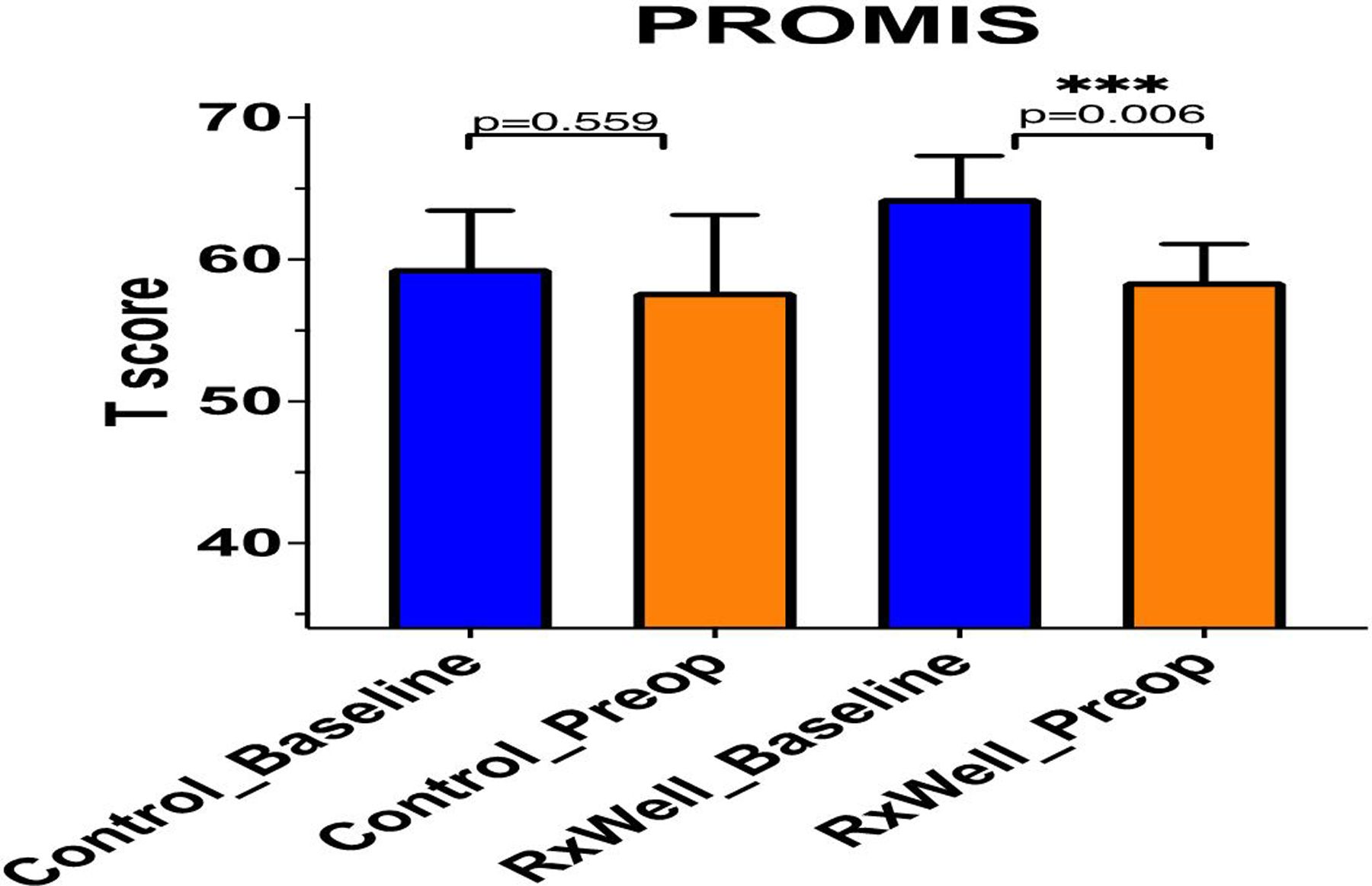
PROMIS T scores of patients schedules to undergo a TKA at the time of randomization (T score >57) and prior to surgery in the control group (no intervention; n=6; p=0.559) and in the treatment group (RxWell^®^; n=5, ***p=0.006). **Note:** (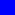) Control_Baseline, RxWell_Baseline; (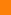) Control_Preop.

PROMIS T scores for patients randomized to the RxWell^®^ group significantly decreased from 64.3 ± 3.0 at the time of randomization to 58.5 ± 2.6 prior to surgery (n=5; p=0.006), whereas no changes in T-scores were recorded in the control group (59.4 ± 4.2 at the time of randomization *vs*. 57.7 ± 6.2; n=6; p=0.559 prior to surgery). Figure 1 presents the PROMIS T scores at the time of randomization (baseline) and prior to surgery (preop) in the control and RxWell^®^ group.

## Discussion

Among patients undergoing TKA, the prevalence of clinically important anxiety or depression is reported to be around 20% [25,26]. Current practice in orthopedics does not include preoperative assessment of mood disorders. Our data suggests by asking patients to complete a PROMIS emotional anxiety short form 8a and/or PROMIS^®^ emotional depression short form-8a questionnaires at least 1 month prior to surgery it is possible in patients with a T score >57 to enroll in a RxWell^®^ program. Such an approach allows normalizing these scores prior to surgery [27–31]. Of a special interest was the fact that these scores did not change in the absence of treatment.

This internet-delivered or mobile phone messaging-based cognitive behavioral therapy programs that was successfully used during the COVID-19 pandemic to control anxiety and depression in patients with chronic pain syndromes [32,33]. Our data suggest that such an approach can be considered in patients undergoing primary unilateral TKA is beneficial [34–38].

## Conclusion

Our data suggest that the use of a comprehensive telemedicine-based digital cognitive behavioral program allows clinicians to effectively control anxiety and depression which is an established cause of postoperative increase in pain and opioid consumption in patients undergoing TKA a month prior to surgery. However, additional studies are required to confirm this concept.
